# 

**Published:** 1887-12

**Authors:** 


### BOOK NOTICES.

Human Culture and Cure.—This is the title of an exhaustive work in six parts, by the widely known author and lecturer, E. D. Babbitt, M.D., D.M., Dean of the N. Y. College of Magnetics.

To make anything like an exhaustive review of part first of this work, which is before us, would consume more space than we are able to allot to such matters in the course of a whole year. We may say however, in a few words, that the work when completed, bids fair to be the crowning one of its author in that field, in which he has already acquired great eminence in this and other countries. Some of the mysteries which were never even approached, much less explored, by the more eminent scientists whose names have become familiar to the students of nature, are explained by Prof. Babbitt who aims to base his conclusions upon first principles, rather than conceded laws, which leave them still a mystery. To this end he begins with simple atoms and traces their union and combination by undeviating laws, into visible forms of matter.

Chapters 17 and 83, wherein an explanation is given of the action of chemical forces in the formation of those abundant chemical compounds upon which all forms of organic life depend, would go far to establish for Prof. Babbitt the claim of a discoverer, in this interesting field of scientific research. We are here shown that it requires the union of two or more contrasting or affinital elements, which naturally flow together to constitute those particles of matter which are apparent to our visual perception. It is indeed this law of affinital attraction which underlies all force, and Prof. Babbitt has turned his critical knowledge of it, especially as regards light, heat, electricity and animal magnetism, to good account in founding the New York College of Magnetics wherein is taught the scientific application of natural means in the treatment and cure of various diseases, which defy ordinary methods.

The work in question is already assured a very large sale, and its influence cannot fail to be highly beneficial, and instructive. For sale at the New York College of Magnetics, 39 West 27th. St. in six parts, each 50 cts.

Woman's Work.—One of the signs of the times is the increasing number of publications specially devoted to the interests of women morally, intellectually socially and politically. There is no plainer mark of the growth of civilization, than the gradual enfranchisement of woman from a state of almost abject slavery to an owner, termed by usage and legal courtesy, a husband, to one of social equality with the male in more enlightened communities.

The headlines of this notice constituted the title of a new monthly, published at Athens, Ga. at the low price of 50 cts. a year. We wish it success.

THE DECEMBER CENTURY, still continues its war articles which are read with great interest by thousands of our citizens. The Life of Abraham Lincoln is approaching the most interesting period in the career of that remarkable statesman; the frontispiece is a portrait of Lincoln from a photograph made about the time of his inauguration. Altogether, the Century is in the fore-front of American monthlies of its class.

WOMAN, is the title of a new, interesting and handsomely illustrated monthly magazine of 72 pp. published by the Woman Publishing Co., at 122 Nassau St., New York City, at $2.75 a year. Its illustrated articles are very interesting. The one on the Astor Library explains that there are now in the three great halls, 1,800 presses of books, each capable of holding 175 volumes, and which if extended in a line, would exceed seven miles in length. May this work meet with deserved good fortune.

THE ALIENIST AND NEUROLOGIST, a quarterly journal of Scientific, Clinical and Forensic Psychiatry and Nemology. Edited by C. H. Hughes, M.D., and associates, St. Louis, Mo., is at hand. Terms $5.00 per ann., or $1.25 a vol. This is a work of 200 pages very handsomely printed and containing special matter of value to Physicians and Surgeons.

We welcome to our table the following publications: *The New Christianity*, Vol. I. No. 1. Philadelphia published every Thursday by the Swedenborg Publishing Association, at $2.00 a year.

THE COSMOPOLITAN for November has been laid upon our table—Its contents include an illustrated article on The Noble Art of Self Defence, by Julian Hawthorne. Schlicht and Field, Publishers, 29 Park Row, N. Y. City, $2.00 a year.

HORTICULTURAL ART JOURNAL, The November issue of this elegantly illustrated monthly is unusually attractive, having four full page colored illustrations. Published by Stecher Lithographic Co., Rochester, N. Y., and clubbed with Hall's Journal of Health at $3.00 per annum.

VICK'S ILLUSTRATED MONTHLY MAGAZINE. James Vick, Seedsman, Rochester, N.Y. Publisher, for November, is resplendent in morning glories besides its usual complement of instructive reading. Terms $1.25 per year.

THE YOUTH'S COMPANION.—The very best youth's paper published in this country, comes out with a thanksgiving number in a handsomely illustrated cover. Weekly, Perry Mason & Co., Boston. $1.75 per annum.

INTERNATIONAL MEDICAL AND SURGICAL SYNOPSIS, published by an Association of the same name, 515 Pine Street, St. Louis, Mo., is replete with valuable information of a class indicated by its title. We have received the October number only.

### TABLE OF CONTENTS.

### 1887.

PAGE.

A Grander System of Human Life....27
A Strange Story from Chicago....89
A Wonderful Medium....138
A Cure for Consumption....139
A Curious Pathological Phenomenon....156
A New Remedy for Burns....157
A Psycographic Experiment....181
Amusements....193
A Natural Healer....198
A Triumph for Pasteur....210
A Curiosum....235
A Remarkable Verification....241
A Case of Trance....273
A Three-time Winner....276
Bathing, its Agency in Therapeutics....150
Book Notices....278
Care of Teeth....16
Children and Pet Animals....207
Clothes that Kill....209
Cancer....249
Concerning the Food We Eat....266
Doctors Disagree....4
Dream Land, Still they Come....64
Drugs and Medicines....86
Dr. R. C. Fisher's Fasting Cure....108
Damp Cellars....229
Dress....232
Defective Speech....233
Dental Surgery....88
Electricity....15
Evils of Water Drinking....90
Epistaxis, or Bleeding of the Nose....152
Equilibrium, the Controlling Force in Nature....182
Electrical Mechanism....252
Fevers—Congestive and Inflammatory....43
Food Adulterations....83
Food Adulterations....103
Food Adulterations....112
Flesh Diet for Children....234
Forewarning....254
God and the Robin....14
How to Retain Health....17
Healthful Breathing....42
Henry Ward Beecher....81
Hygiene, Its Agency in Therapeutics....84
How a Star is Weighed....134
Healing by Laying on of Hands....176
Heloise....211
Household....19
Household....45
Household....69
Household....93
Household....116
Household....143
Household....165
Household....189
Household....213
Household....237
Household....261
Household....280
Horsford's Acid Phosphate....61
How Ben. Butler got Rich....284
Injurious and Adulterated Beverages....77
Injurious and Adulterated Beverages....102
Ill Health from Over Eating....195
Influence of Drugs, &c....231
Instinct of Animals....223
Language of the Hand....154
Little Busy Bee....188
Laws of Health....200
Libraries of the World....205
Liability of Physicians....208
Literary Thefts....232
Last Words....265

PAGE.

Molecules and Atoms....113
Mind Reading....170
Milk and Its Products....184
Mesmerism Forty Years Ago....225
Mind Cure....255
Miscellaneous....21
Miscellaneous....47
Miscellaneous....71
Miscellaneous....95
Miscellaneous....118
Miscellaneous....141
Miscellaneous....167
Miscellaneous....191
Miscellaneous....215
Miscellaneous....239
Miscellaneous....263
Miscellaneous....282
Microbes that Endanger Health....267
Natural Gas....220
Oxygen—Its Agency in Therapeutics....10
Oxygen—Its Agency in Therapeutics III....34
Oxygen—Its Agency in Therapeutics....53
Our Prospects and Purposes....217
Over the Dasies....236
Phrenology....121
Pyscometry....145
Physiognomy of the Nose....148
Physiognomy Illustrated....155
Physical Decay of Cities....158
Pre-Digested Foods....137
Prohibition....218
Progress....277
Quinine....82
Rheumatism....91
Relation of Exercise to Health....162
Red and White....163
Retrospect....283
Somnambulism....23
Somniloquism....49
Sore Eyes....60
Singular Pre-Natal Affliction....66
Surgery Extraordinary....87
Strange Admonition of Death....111
Some Forebodings of Incipient Insanity....114
Spiders....159
Samuel Hahnemann....124
Seeing by the Interior Sense....173
Spurious Wines....186
Spirit Likeness....203
Starving the Teeth....206
Shakers and Shakerism....244
Stand up for the New and the True....270
Schools in Medicine....272
The New Year....1
The Electric Light....3
The Skin, its Correlations and Functions....62
The Heart, its Functions....63
The Occult Forces....73
The Prayer Cure Practically Applied....80
The Occult Forces....97
Transplanting an Eye....129
The Voice of a Friend....140
The Vassar Girls....159
The Comparative Energy of Nutrients....179
The Spirit Form....204
Treatment of the Hair....230
The Olive Tree....256
The Seybert Commission....257
The Little Maid and the Butterfly....259
Use of Toilet Soap....253
What are we Drinking....34
Woodlands and Malaria....56
Water, Its Agency in Therapeutics....106
What Every Girl Ought to Learn....251
What of the Potato....274

